# Soluble ADAM10 has a decreased activity compared to membrane‐bound ADAM10 isoforms

**DOI:** 10.1002/alz70855_105411

**Published:** 2025-12-24

**Authors:** Vanessa Alexandre da Silva, Marina Mantellatto Grigoli, Angelina Maria Fuzer, Patricia Regina Manzine, Sabrina Dorta de Oliveira, Marcia Regina Cominetti

**Affiliations:** ^1^ Universidade Federal de São Carlos, São Carlos, SP, Brazil; ^2^ Federal University of São Carlos, São Carlos, São Paulo, Brazil; ^3^ Federal University of São Carlos, São Carlos, SP, Brazil

## Abstract

**Background:**

Alzheimer's disease (AD) pathology involves the accumulation of amyloid‐beta (Aβ) peptides into senile plaques generated by Amyloid Precursor Protein (APP) cleavage by β‐secretases. When APP is cleaved by ADAM10 instead, neuroprotective fragments are released. ADAM10 possesses three isoforms: a zymogen (proADAM10), a proteolytically active mature (mADAM10), and a soluble (sADAM10) isoform. Our group has shown that sADAM10 levels and activity are altered in the plasma of persons with AD. Thus, we aimed to investigate the levels and activity of ADAM10 isoforms in neuron‐like cells to understand their central functioning.

**Methods:**

SH‐SY5Y cells were differentiated into neuron‐like cells using retinoic acid. The media was collected, and the cells were fractionated into cytoplasmic, membrane‐ bound, and nuclear protein‐rich portions. Western blotting (WB) experiments were performed on each fraction to detect ADAM10 isoforms and assess fraction purity using anti‐GAPDH (cytoplasm), anti‐VDAC (membranes), and anti‐Lamin A/C (nuclei) antibodies. Antibodies targeting the *N*‐terminal and C‐terminal regions of ADAM10 were used to detect the soluble and membrane‐bound isoforms, respectively. Additionally, enzymatic activity assays were conducted.

**Results:**

The characterization of the fractioning process and preliminary results of the activity assays show feeble sADAM10 activity in the cell medium, compared to mADAM10 in the membrane and cytoplasm fractions and recombinant ADAM10 (*p* < 0.001).

**Conclusion:**

These preliminary results show that sADAM10 has feeble activity in neuron‐like cells, which agrees with our previous findings, validating the potential use of plasma ADAM10 as a blood‐based AD biomarker.

**Funding**: São Paulo Research Foundation (FAPESP) grants #2023/00449‐0; #2023/18020‐0 and 2021/01863‐9

